# A Multimodal Approach to the Management of Neuroendocrine Tumour Liver Metastases

**DOI:** 10.1155/2012/819193

**Published:** 2012-02-15

**Authors:** Ron Basuroy, Rajaventhan Srirajaskanthan, John K. Ramage

**Affiliations:** ^1^Department of Gastroenterology and Hepatology, Basingstoke and North Hampshire Foundation Trust, Basingstoke RG24 9NA, UK; ^2^Neuroendocrine Tumour Service, Institute of Liver Studies, King's College Hospital, London SE5 9RS, UK

## Abstract

Neuroendocrine tumours (NETs) are often indolent malignancies that commonly present with metastatic disease in the liver. Surgical, locoregional, and systemic treatment modalities are reviewed. A multidisciplinary approach to patient care is suggested to ensure all therapeutic options explored.

## 1. Introduction

Neuroendocrine tumours (NETs) are uncommon tumours that can arise anywhere within the body, but predominantly from the gastroenteropancreatic tract. Recent epidemiological evidence suggest that the incidence of all NETs is approximately 3–5 per 100,000 population per year with a prevalence of 35 per 100,000 population because of slow tumour growth [1, 2]. Though most NETs are nonfunctional, others secrete peptide hormones that can cause clinical syndromes, like flushing, diarrhoea, bronchospasm and palpitations seen with carcinoid syndrome. The majority of these tumours are indolent, slow growing malignancies, commonly presenting with metastatic disease. The most common site of distant metastases is the liver. Consequently, many therapies are focused at treating the primary and also the metastatic disease in the liver. Due to the indolent nature of most of these tumours, the 5-year survival of patients with metastatic disease at presentation is approximately 50%. There may have been some improvement in survival from medical and surgical therapies. New molecular-targeted therapies and an aggressive surgical approach to resection of primary and secondary tumours show benefit.

This paper focuses on management of liver metastases of NETs and covers both surgery, locoregional, and systemic therapy. In general, local therapies to the liver should be considered first if disease is confined to the liver. This allows systemic therapies to be given at a later stage if there is extrahepatic spread. Results for liver-directed and systemic therapy of neuroendocrine tumour liver metastases are summarised in Tables 1 and 2, respectively.

## 2. Liver-directed Therapies

Consensus guidance recommends surgery for liver metastases in well-differentiated NETs if complete resection or debulking of <90% tumour load is feasible [45]. However, intended curative surgery is only possible in less than 10% of patients who are diagnosed with hepatic metastases at presentation [3, 46–49]. The distribution of liver metastases affects survival; solitary metastases, isolated metastatic bulk with smaller accompanying deposits, and disseminated metastatic spread have 5-year survival rates of 100%, 84%, and 51%, respectively [50].

An aggressive surgical approach to resecting liver metastases is supported by significantly improved actuarial survival in series compared to nonrandomised controls [3–5]. A number of different surgical approaches are available depending on the distribution of metastases. If primarily unilobar metastases are present, a one-step approach can be adopted. In these cases, resection of the primary plus liver resection can be performed. A two-step surgical approach to bilobar metastases from luminal NETs with resection of the primary, limited resection of left liver lobe metastases, and right portal vein ligation followed by right hepatectomy has been proposed [6]. Overall survival and disease-free rates at 5 years were 94% and 50% with this approach. Other series report a range of overall survival and disease-free rates [7–9, 51]. A significant improvement in 3-year survival for surgical resection over medical treatment or embolisation has been demonstrated in a study limited by bias. [10]. The completeness of resection, in particular resection margin involvement, is thought to be more important than the number, localization, and size of liver metastases [7, 52, 53]. Histological grade and extrahepatic disease are predictive of overall survival [54, 55]. Disease has been shown to recur in 78–94% of patients at 5 years [3, 8, 11]. 

After surgery, patients with functioning tumours have prolonged partial or complete symptomatic response rates that can contribute to improved quality of life [11, 56, 57]. Patients with carcinoid tumours have reduced biomarkers (e.g., Chromogranin A and urinary 5-HIAA) after surgery that correlate with symptom relief and disease control [3, 58]. Some rarer functioning syndromes, like those related to PTHrP or VIP secretion, can be improved by debulking surgery [59].

There is no evidence from randomised clinical trials supporting liver surgery, either for curative resection or for debulking in nonresectable disease, over other treatment modalities. Liver surgery only achieved significance in improving survival in univariate but not multivariate analysis [60–62]. Neoadjuvant strategies for downsizing liver metastases or adjuvant chemotherapy following hepatic resection have not yet been subject to controlled clinical trials [63–65].

### 2.1. Surgery to Primary Tumour in Metastatic NETs

Recent guidelines recommend resection of the primary tumour and mesenteric lymph nodes in jejunum/ileum NETs [66–68]. Tumour mass reduction or debulking of primary jejunal and ileal NETs reduces the possibility of bowel ischaemia and obstruction from tumour and mesenteric lymph nodes mass effect even in the context of liver metastases. Resection of the primary tumour has been shown to be an independent positive predictor of survival (*P* = 0.015) and associated with a significantly longer survival than no resection (median survival 7.4 versus 4.0 years; *P* < 0.01) [62, 69]. Successful resection of mesenteric metastases and the desmoplastic reaction around the primary site are also associated with a significantly longer survival. Significant reductions in tumour-related symptoms are also seen after primary and mesenteric lymph node resections.

Aggressive surgery to primary tumours and resectable liver metastases in pancreatic NETs is recommended [67, 70]. Resection of pancreatic NETs has been suggested to be associated with significantly improved survival compared to those who did not undergo resection (114 months versus 35 months; *P* < .0001) though significant biases may exist in this study [71]. This survival benefit was demonstrated for patients with localized, regional, and metastatic disease with an adjusted odds ratio of 0.48. Independent predictors of survival after resection of pancreatic NETs include age, grade, presence of distant metastases, tumour functionality, and type of resection [72]. Current guidelines do not recommend surgery to the primary pancreatic tumour in patients with unresectable liver metastases [70, 73].

### 2.2. Transplantation

The role of orthotopic liver transplant is controversial given the demand for donor organs and a lack of clear selection criteria [74]. Patients with debilitating and poorly controlled hormonal syndromes from small intestine or pancreatic NETs are considered for transplantation as symptom relief is seen in 90% of patients following surgery [12, 13, 75–78]. Five-year recurrence-free rates vary from 25–50%. Overall five-year survival rates are around 50% but vary according to patient selection [13, 14, 79, 80]. Patients presenting with duodenal or pancreatic NET in association with hepatomegaly have poorer outcomes (12% versus 68% five-year survival rates) [14]. The presence of extensive extrahepatic tumour resected at the time of transplantation is associated with poorer median and five-year survival rates of ten months and 30%, respectively [12]. Important selection criteria include well-differentiated tumours, low proliferation rate (Ki-67 < 10%), and regular E-Cadherin staining [81, 82]. The Milan criteria for transplantation include age less than 55 years, low grade carcinoid NET, limited metastatic disease in the liver (<50%), previously resected tumours drained only by the portal system (pancreas and mid gut origin NETs), and stable disease for 6 months [83]. Combination treatment with chemotherapeutic agents, chemoembolisation, systemic radiopeptide treatment, and aggressive surgery for recurrence may lead to improved survival rates [84–86].

### 2.3. Embolisation

NET liver metastases are highly vascular with an arterial supply that if occluded will lead to ischaemia and necrosis. Normal tissue is supplied from the portal vein and preserved during embolisation of hepatic arteries. A catheter is guided to the hepatic artery or branch and material (gelfoam powder, microembospheres, and polyvinyl alcohol particles) released to occlude the vessel in bland embolisation. In chemoembolisation, cytotoxics (like cisplatin, mirplatin, gemcitabine, doxorubicin, streptozocin, and 5-FU) are injected prior to arterial embolisation in order to achieve higher concentrations and prolonged action in necrotic tissue [87–89]. Contraindications to embolisation include occlusion of the portal vein, severe liver dysfunction, and presence of biliary anastomosis. Relative contraindications include tumour burden, renal impairment, and heart disease (including carcinoid heart disease) [90, 91]. A postembolisation syndrome may occur with abdominal pain, vomiting, fever, and rise in transaminases.

Vascular occlusion can achieve reduced hormonal symptoms from NET syndromes, reduced tumour burden, and improved survival in patients who have tried medical therapy and who are not suitable for surgical resection [92–95]. Sequential hepatic artery occlusion can offer prolonged palliation for responsive patients even if performed later in their clinical course [90, 96, 97].

Median survival rates after transarterial embolisation (TAE) or chemoembolistaion (TACE) in patients with liver metastases is over 3 years with progression-free survival (PFS) of around 18 months [15–18, 98–100]. Clinical response rates of over 90% are seen following treatment [91]. Intact primary tumour, extensive liver disease, and bone metastases are associated with worse outcomes. Embolisation of nonresectable liver metastases often results in disease regression in patients with carcinoid or pancreatic NETs [17, 19]. TACE appears to benefit patients with pancreatic NETs while TAE benefits those with ileal NETs [20]. A small randomized study of TAE versus TACE in all liver NETs has shown no difference in time to progression [101].

### 2.4. Radiofrequency Ablation (RFA)

RFA of oligonodular liver metastases (fewer than 8) of less than 5 cm can result in symptomatic response in 70–80% of patients with hormonal syndromes for as long as 24 months [21, 63, 102, 103]. Electrical energy is delivered to tissues via a catheter, inserted percutaneously or laparoscopically, which leads to heating and cell death [104, 105]. Microwave RFA can reduce time required for this procedure. RFA can play an important role in the treatment of carcinoid metastases not suitable for surgical resection and refractory to TAE, improving symptom control, reducing octreotide dependence, and slowing progression in patients [106–108]. Limitations to using RFA include increased numbers and size of liver metastases as well as the detrimental cooling effect of blood flow from neighbouring blood vessels. Local recurrence has been identified in 21.7% of tumours on CT scans with a mean follow-up of 17 months. Recurrence can be predicted by tumour type and size, ablation margin, and blood vessel proximity [103, 109]. Median survival after starting RFA treatment is 3.9 years [21]. Although RFA may play a promising role in the treatment of liver metastases from NETs, its effect on survival and tumour progression needs to be explored in larger studies. In particular, studies are needed comparing surgical resection with RFA.

### 2.5. Selective Internal Radiation Therapy (SIRT)

Radioembolisation of liver metastases can be achieved with Yttrium-90 resin microspheres in patients with disseminated and inoperable liver disease even if previous TAE or TACE has taken place [22, 110]. (90 Y) microspheres are injected through a percutaneously placed hepatic artery catheter via the femoral or brachial artery. Contraindication to SIRT is similar to those of bland embolisation, vascular involvement such as portal vein thrombosis, severe liver dysfunction, and large tumour burden. Long-term radiologic and biological responses can be achieved with radioembolisation with partial or complete response seen in 63% [22, 23]. Median survival varies from 36 to 70 months [23, 24]. Prognostic factors include radiographic response to treatment, tumour grade, and presence of extrahepatic disease. Patients with hepatic tumour burden of 20–50% by volume, well-differentiated tumour, female gender, and no extrahepatic disease benefit most from treatment [25]. There is no randomized evidence that radiologic and symptom response rates following SIRT are different from those seen with TACE and TAE.

## 3. Systemic Therapies

### 3.1. Biological Therapy

Over 70% of NETs express cell-surface somatostatin receptors that are targeted by synthetic somatostatin analogues. Patients with functional NETs can derive significant symptomatic benefit from the use of somatostatin analogues that suppress the secretion of peptide hormones. Octreotide can provide symptomatic response in up to 85% of patients and biochemical response in up to 70% of patients within weeks of commencement [111, 112]. Patients with NETs undergoing interventional procedures can experience severe symptoms related to the release of vasoactive hormones, like serotonin, that can cause a carcinoid crisis with bronchospasm, tachycardia, and labile blood pressure. This can be ameliorated through the use of octreotide infusions before, during, and after interventional procedures.

Some groups have reported an antiproliferative property of somatostatin analogues [26, 27, 112, 113]. Octreotide LAR has been found to significantly lengthen the time to tumour progression compared to placebo injections (14.3 versus 6 months resp.) [28]. The benefit was seen in both functionally active and inactive tumours. Patients with low hepatic tumour load and resected primary tumour benefited the most from treatment with octreotide LAR. Overall, survival was not an endpoint of this study, consequently; survival benefit from the use of somatostatin analogues has not been confirmed.

Interferon alpha 3–5 megaunits 3–5 times per week have been used with some symptomatic response, but no clear reduction in tumour size or survival benefit [29–31, 114, 115]. Interferon alpha should be considered as second-line biological therapy after somatostatin analogues.

### 3.2. Chemotherapy

Systemic chemotherapy has a role in the treatment of pancreatic and high grade NETs. Patient selection and individualized treatment are required to minimize toxicity, maximize response, and improve overall quality of life. The degree of differentiation and tumour grade of NETs can guide management [116, 117]. Poorly differentiated and high-proliferative tumours (from histological grading like Ki-67 and mitotic index) behave more aggressively but are more sensitive to cytotoxic therapy than well-differentiated and low-proliferative tumours (Ki-67 < 10%) [33]. Objective response to chemotherapy varies between 25–78% with progression-free periods between 4–22 months [32, 34, 37, 118–124]. Therefore, it is essential to ensure that chemotherapy is offered to patients who are likely to respond; those with pancreatic NETs, aggressive phenotypes, and high proliferation rates [125]. Biochemical and radiological progression in asymptomatic patients identifies those with rapidly progressive disease and an aggressive phenotype [67]. Response to cytotoxic therapy can be established from radiological-quantified reduction in tumour size, improved biochemical markers as well as improvements in quality of life as measured by health questionnaires [126–128].

Single-agent chemotherapy is seldom used because of limited response rates, toxicity, and poor survival rates. Newer agents like paclitaxel, temozolomide, topotecan, and gemcitabine are not markedly better than older agents like streptozocin, dacarbazine, 5 flourouracil, and doxorubicin when used as monotherapy [121, 126, 129–135]. In patients with pancreatic NET, combination chemotherapy with streptozocin and doxorubicin is superior to streptozocin and 5FU in terms of response rates, time to progression, and overall survival [32, 136, 137]. Response rates from streptozocin and doxorubicin combination treatment vary between 30–70% [33–35, 138]. Recently, a retrospective analysis of capecitabine and temozolomide combination chemotherapy has demonstrated good response rates, superior to traditional streptozocin-based chemotherapy [36]. In 30 patients treated with capecitabine and temozolomide, response rates of 70%, progression-free survival of 18 months and overall survival of 92% at 2 years were observed. However, streptozocin-based therapy remains the standard chemotherapy regime for pancreatic NETs given the lack of data from randomised trials demonstrating benefit from other regimes [36, 116, 123, 139, 140]. Poorly differentiated or anaplastic NETs respond to a combination of cisplatin and etoposide, a regime used in small cell lung cancer [37, 118–120]. Despite chemotherapy, the prognosis remains poor in this group with a 2-year survival between 20–30%.

### 3.3. Molecular-Targeted Therapies

Novel systemic agents target the molecular mechanisms that are implicated in the pathogenesis of NETs [141, 142].

Sunitinib, a multitargeted tyrosine kinase inhibitor, has activity against a range of molecular targets, including VEGF receptors and platelet-derived growth factor receptors, and has been shown to have antitumour activity in pancreatic NETs [143]. Median PFS is significantly longer in patients treated with sunitinib over placebo (11.4 versus 5.5 months) [38]. Objective response rates and overall survival are also improved with sunitinib treatment. Frequent adverse events encountered include diarrhoea, nausea, vomiting, asthenia, and fatigue.

Everolimus, an oral inhibitor of mammalian target of rapamycin (mTOR), has activity against pancreatic NET tumours through a mechanism of cellular apoptosis and antiangiogenesis [144, 145]. Median PFS is significantly longer in those treated with everolimus over placebo (11 versus 4.6 months) [39]. Severe adverse events like hyperglycaemia and anaemia were rare, with stomatitis, diarrhoea, and fatigue are more commonly seen.

Vascular endothelial growth factor (VEGF) is overexpressed in NETs and targeted by the ligand monoclonal antibody Bevacizumab [40, 146, 147]. There are reports of clinical benefit when combined with existing chemotherapy treatments [148, 149].

### 3.4. Peptide Receptor Radionuclide Therapy (PRRT)

Somatostatin receptors subtype 2 are expressed in the majority of NETs and confirmed through uptake in octreotide scintigraphy or somatostatin-based PET imaging [150–152]. Beta-emitting 90 Y- and 177 Lu-labeled somatostatin analogues have been studied in patients with metastatic and inoperable disease [41, 42, 153–156]. The majority of patients develop stable disease with the average time to progression of 40 months from commencing therapy. Partial and complete objective responses are seen in up to 30% of patients with median PFS of over 2 years [43, 157]. From diagnosis, there is a survival benefit of 40–72 months compared to historical controls [44]. Predictive factors include high tumour uptake on scintigraphy and limited liver metastases. Adverse events include bone marrow and liver toxicity as well as radiation-induced lose of renal function and gastrointestinal disturbance from the use of renoprotective agents [158, 159]. The addition of radiosensitisers like gemcitabine and capecitabine to PPRT may improve clinical outcomes [160, 161]. Alpha-emitting isotopes, such as actinum-225 (225Ac), have a higher cytotoxic activity than beta emitters and may be used in PRRT [162].

MIBG scans are also used to identify patients with metastatic NETs. 131 I-MIBG therapy is associated with significantly improved 5-year survival rates of 85% (nonrandomized studies) as well as marked symptomatic and hormonal improvement [163–165]. Symptomatic response predicts improved survival.

## 4. Conclusion

**Figure 1:**
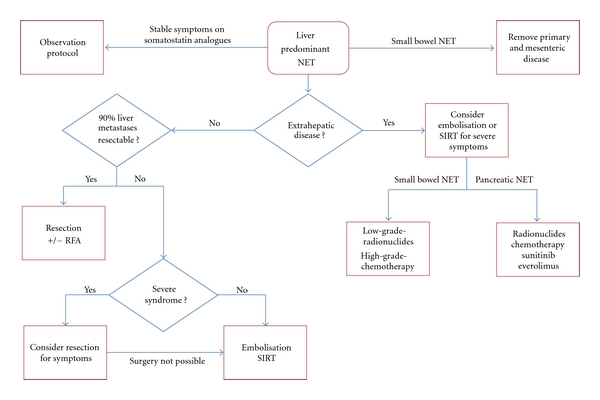


There are a number of treatment modalities available in the management of neuroendocrine tumour liver metastases with a treatment algorithm outlined in Figure 1. Proactive surgical resection, with curative intent or for debulking (cytoreduction), has been shown to improve outcomes and should be pursued initially. In patients with more advanced disease or not amenable to surgical resection, locoregional therapies, like embolisation and SIRT, offer improved outcomes and may downstage disease. Newer systemic therapies, in particular PRRT and molecular targeted therapies, can play a role in patients with extrahepatic and progressive disease. Although there is a lack of robust evidence-based data in the management of patients with metastatic NETs, the future appears more positive with the range of treatment options available. An individualized approach to patient care is needed given the breadth of symptoms and disease, the lack of a validated treatment pathway as well as the indolent nature of NETs. Patient care should be managed under the auspices of a multidisciplinary team to ensure that all treatment options are explored both at diagnosis and follow-up.

## Figures and Tables

**Table 1:** Summary of results for liver-directed therapy of neuroendocrine tumour liver metastases.

Modality author [Ref]	Intervention	Number of patients	Overall survival (5 years)	Median survivals (months)	Progression/disease-free survival	Clinical response	Biochemical response	Radiological response
Liver surgery								

Sarmiento et al. [3]	Resection	170	61%					
Touzios et al. [4]	Resection ± ablation	18	72%	>96				
Grazi et al. [5]	Resection	19	92% (4 yrs)					
Kianmanesh et al. [6]	Resection	23	94%		50% (5 yrs)			
Gomez et al. [7]	Resection	18	86%		66% (5 yrs)			
Scigliano et al. [8]	Resection	41	79%		3% (5 yrs)			
Osborne et al. [9]	Cytoreduction	61		Curative-50 (mean)				
				Palliative-32 (mean)				
Musunuru et al. [10]	Resection ± ablation	13	83% (3 yrs)					
Mayo et al. [11]	Resection ± ablation	339	74%	125				

Liver transplantation								

Lehnert [12]		103	47%					
Olausson et al. [13]		15	90%		20% (5 years)			
Le Treut et al. [14]		85	47%					

Embolisation								

Ho et al. [15]	TAE or TACE	46		42	18 months			
Ruutiainen et al. [16]	TACE	57	50%		35% (3 yrs)			
Strosberg et al. [17]	TAE	84		36				
Dong and Carr [18]	TACE	123	36%	39 (mean)				
Ruszniewski et al. [19]	TACE	24				73%	57%	33%
Gupta et al. [20]	TAE or TACE	69 (Carcinoid)			22 months			67%
		54 (Pancreatic)			16 months			35%

RFA								

Mazzaglia et al. [21]		63		46 (after RFA)		70%		

SIRT								

King et al. [22]		37		29 (mean)		55%	43%	50%
Kennedy et al. [23]		148		70				63%
Cao et al. [24]		58	47% (3 yrs)	36				34%
Saxena et al. [25]		48		35				55%

**Table 2:** Summary of results for systemic therapy of neuroendocrine tumour liver metastases.

Modality author [Ref]	Intervention	Number of patients	Overall survival (5 years)	Median survivals (months)	Progression/disease-free survival	Clinical response	Biochemical response	Radiological response
Biological Therapy								

Ducreux et al. [26]	Lantreotide	46						5%
Aparicio et al. [27]	Octreotide	35			11 months			3% (57% stabilised)
Rinke et al. [28]	Octreotide LAR	85			14.3 months			67% stabilised
Oberg and Eriksson [29]	IFN *α*	111		>80	34 months	68%	42%	15% (39% stabilised)
Arnold et al. [30]	Octreotide ± IFN *α*	109		32 versus 54 (combined)				1.9% (27% stabilised)
Fjällskog et al. [31]	Somatostatin ± IFN *α*	16					62.5%	19%

Chemotherapy								

Moertel et al. [32]	STZ + doxorubicin	36		26	20 months			69%
	STZ + 5FU	33		18	6.9 months			45%
Turner et al. [33]	5FU + cisplatin + STZ	79		31.5	9.1 months			33%
Sun et al. [34]	STZ + doxorubicin	85		15.7	4.5 months			15.9%
	STZ + 5FU	78		24.3	5.3 months			16%
Kouvaraki et al. [35]	5FU + doxorubicin + STZ	61	74% (2 yrs)		41% (2 years)			
Strosberg et al. [36]	Temolozomide + capecitabine	30	92% (2 yrs)		18 months			70%
Moertel et al. [37]	Etoposide + cisplatin	18		19	8 months			67%

Molecular-targeted therapy								

Raymond et al. [38]	Sunitinib	171			11.4 months			9.3%
Yao et al. [39]	Everolimus	410	34% (1.5 yrs)		11 months			
Yao et al. [40]	Bevacizumab	44			95% (18 weeks)			

PPRT								

Cwikla et al. [41]	DOTATATE Y-90	60		22	17 months	72%		23%
Kwekkeboom et al. [42]	177Lu-octreotate	131			>36 months			28%
Pfeifer et al. [43]	Y-DOTATOC or 177Lu-DOTATOC	69			29 months			23.6%
Kwekkeboom et al. [44]	177Lu-DOTA 0,Tyr3	310		46	40 months			30%
